# GLUT1 and CAIX as intrinsic markers of hypoxia in bladder cancer: relationship with vascularity and proliferation as predictors of outcome of ARCON

**DOI:** 10.1038/sj.bjc.6601260

**Published:** 2003-09-30

**Authors:** P J Hoskin, A Sibtain, F M Daley, G D Wilson

**Affiliations:** 1 CR UK Tumour Biology and Radiation Therapy Group, Mount Vernon Cancer Centre, Rickmansworth Road, Northwood, Middlesex HA6 2RN UK; 2 Gray Cancer Institute, Mount Vernon Hospital, Northwood, Middlesex HA6 2RN, UK

**Keywords:** hypoxia, bladder cancer, pimonidazole; carbonic anhydrase IX, glucose transporter protein 1 (GLUT1)

## Abstract

Glucose transporter-1 protein (GLUT1) and carbonic anhydrase IX (CAIX) are regulated by hypoxia inducible factor-1 (HIF-1) and have been studied as putative intrinsic cellular markers for hypoxia. This study directly compares CAIX and GLUT1 with pimonidazole binding in a prospective series of bladder cancer patients and also studies the prognostic significance of the markers, in combination with vascularity and proliferation, in a retrospective series of bladder cancer patients treated in a phase II trial of radical radiotherapy with carbogen and nicotinamide (ARCON). A total of 21 patients with a diagnosis of transitional cell carcinoma of the bladder received 0.5 g m^−2^ pimonidazole. Serial tumour sections were stained for pimonidazole, GLUT1 and CAIX and compared. Tissue sections obtained from a series of 64 patients previously treated for invasive bladder cancer using ARCON were stained for GLUT1 and CAIX together with Ki-67 and CD31/34. There was a good geographical colocalisation of both intrinsic markers with pimonidazole and a highly significant agreement in individual patients; correlation coefficients were 0.82 (*P*=0.0001) for GLUT1 and 0.74 (*P*<0.0001) for CAIX. In both series of patients, the intrinsic hypoxia markers were highly correlated with each other and a correlation with proliferation was also evident in the retrospective study. In univariate and multivariate analyses, GLUT1 and CAIX were independent predictors for overall and cause specific survival. The hypoxia markers did not predict for local control or metastases-free survival although higher Ki-67 indices showed a trend towards local failure. The data suggest that both hypoxia modification and accelerated treatment may be valid treatment options in bladder cancer.

Bladder cancer is common accounting for over 12 000 cases each year in the UK. The muscle-invasive form of the disease is commonly treated with either radical cystectomy or radical radiotherapy. The significance of hypoxia in bladder cancer has been difficult to study due to the limitations of access to oxygen probe-based technology. Recently, we have shown that many bladder tumours are hypoxic using prebiopsy administration of pimonidazole and immunohistochemical staining for bound pimonidazole fragments after nitroreductase-induced breakdown in hypoxic areas (Wykoff *et al*, 2000). The disadvantage of the use of pimonidazole as a marker of hypoxia is the need to administer the drug several hours prior to biopsy and thus an intrinsic immunohistochemical marker of hypoxia would have considerable advantages over this extrinsic method.

There are several putative intrinsic markers of hypoxia under investigation whose common theme is regulation by hypoxia inducible factor-1 (HIF-1) (Semenza, 2000). Of these, two proteins, carbonic anhydrase IX (CAIX) and glucose transporter-1 protein (GLUT1), have received much recent attention (Airley *et al*, 2001,Airley *et al*, 2003; Chia *et al*, 2001; Giatromanolaki *et al*, 2001; Loncaster *et al*, 2001; Olive *et al*, 2001; Swinson *et al*, 2003). In an initial series of bladder cancers, CAIX was found to colocalise with pimonidazole (Wykoff *et al*, 2000). More recently, in superficial and muscle-invasive bladder cancer there was overlap in the expression of vascular endothelial growth factor (VEGF) and CAIX, CAIX being more widespread (Turner *et al*, 2002). It was noted that the expression of CAIX was absent within 80 *μ*m of microvessels and a similar experience has now been reported with GLUT1 in cervical cancer (Airley *et al*, 2001).

This investigation consists of two arms. The first utilised a series of prospective bladder cancer patients who were studied with pimonidazole administration to establish the correspondence between cellular hypoxia and CAIX and GLUT1 distributions. Second, tumour specimens from a cohort of patients with detailed clinical outcome treated in a phase II trial of carbogen and nicotinamide (Hoskin *et al*, 1999) in the radical radiotherapy of bladder cancer, have been reviewed and stained with GLUT1, CAIX, Ki-67 and the combined vascular markers CD31 and CD34. The results of this have been explored in relation to treatment outcome.

## MATERIALS AND METHODS

### Patients and treatments

The prospective cohort comprised 21 patients with a diagnosis of transitional cell carcinoma of the bladder who were to undergo definitive transurethral resection of bladder tumour (TURBT). With appropriate approval from the Local Ethics Committee for Mount Vernon Cancer Centre, patients received 0.5 g m^−2^ pimonidazole (Hydroxyprobe-1™, Natural Pharmacia Inc., Belmont, MA, USA) administered in 100 ml of normal saline by intravenous infusion over 15 min, 8–18 h prior to biopsy. Tumour samples taken at TURBT were fixed in formalin, protected from light and stored at 4°C.

The retrospective series of patients had been treated at Mount Vernon Hospital in a phase II trial of radical radiotherapy with carbogen and nicotinamide. The patients received 50–55 Gy in 20 daily fractions over 4 weeks with carbogen breathing delivered at a rate of 15 l min^−1^ through a sealed face mask and a closed breathing system with a one-way valve, started 5 min prior to radiotherapy and maintained throughout treatment; only four patients failed to tolerate the whole course of carbogen breathing. A total of 33 patients also received nicotinamide (80 mg kg^−1^) given orally at least 1 h after eating and 1.5 h prior to radiotherapy; 31 patients did not receive nicotinamide either due to coexisting vascular disease or patient refusal. Approval from the Local Ethics Committee for Mount Vernon Cancer Centre was granted to approach these patients for permission to use samples of their stored original biopsy material for further analysis. A total of 64 samples was finally obtained and analysed. The characteristics of these patients are shown in Table 1

**Table 1:** Patient characteristics

	**Prospective pimonidazole study**	**Retrospective carbogen patients**
Age (years)	70 (49–87)	73 (38–88)
Median(range)		
*Stage*		
T1	3	6
T2	5	19
T3	12	38
T4	1	1
		
*Grade*		
G1	1	2
G2	10	17
G3	10	55
		
Total	21	64

.

### Immunohistochemistry

In the prospective study, pimonidazole, CAIX and GLUT1 were stained on serial sections using single-staining procedures. Staining for pimonidazole both alone and in combination with proliferation and vascular markers have been reported separately (Hoskin *et al*, 2003 (submitted) Briefly, after dewaxing and rehydration, the sections were pretreated with 0.01% Pronase (Sigma Poole, Dorset UK) in PBS pH 7.8 at 37°C for 10 min. Endogenous peroxidase was blocked using Dako peroxidase block for 5 min and then Dako protein block was applied for 5 min after washing in tap water. Mouse anti-pimonidazole IgG1 monoclonal antibody (Clone Hypoxyprobe™-1MAb1: Natural Pharmacia Inc.) was diluted 1/100 in Tris-buffered saline (TBS, 0.01 M Tris-HCl, 0.14 M NaCl, pH 7.6) and incubated for 30 min at room temperature, this was followed, after washing, by Envision HRP Mouse polymer (Dako High, Wycombe, Bucks, UK) for a further 30 min. After washing, diaminobenzidine (DAB) substrate (7.5 mg DAB (Vector, Peterborough UK), 10 ml 0.1 M Tris buffer, 1 drop 3% H_2_O_2_) was added for 5 min. The slides were rinsed in TBS and then running tap water and placed briefly in Mayer's haematoxylin and mounted in DPX (Merck 360294 H). GLUT1 required microwave pretreatment in 10 mM citric acid pH 6.0 on high power for a total of 12 min (4 × 3 min). CAIX required no pretreatment. The remainder of the staining procedures were the same, the anti-GLUT1 antibody (Dako Hyh, Wycombe, Bucks, UK) was diluted at 1/200 and anti-CAIX (a gift from Adrian Harris, Oxford) was used at a dilution of 1/50.

In the retrospective study to maximise the potential information, each cohort of patients was examined using serial sections and a series of double-staining procedures that consisted of either CAIX or GLUT1 in combination with either Ki-67 or CD31/CD34. The combinations of substrates were governed by the Envision Alkaline Phosphatase (AP) polymer that was only available in anti-mouse form. Therefore, as the GLUT1 and Ki-67 antibodies were polyclonal, the following combinations were used: CAIX (AP)/Ki-67 (HRP), CAIX (HRP)/CD31 (AP) and GLUT1 (HRP)/CD31 (AP). As the CAIX antibody required no pretreatment, it was incubated first when in combination using either Envision horseradish peroxidase (HRP) Mouse polymer (Dako) for 30 min followed by DAB substrate for 5 min (when used with CD31) or Envision AP (Dako) for 30 min followed by the addition of the AP Red substrate (Vector, Peterborough UK) for 5–10 min (when used with Ki-67). After washing, microwave pretreatment in10 mM citric acid pH 6.0 on high power was performed for a total of 12 min (4 × 3 min) Either rabbit anti-human Ki-67 antibody (Dako) diluted 1/200 or mouse anti-CD31/CD34 antibody cocktail (Dako M0823/M7080) diluted 1/50 and 1/100 was incubated in TBS for 1 h at room temperature. Then, the appropriate Envision polymer and substrate was added as described above to visualise the second antigen. The sections were rinsed in TBS and washed well in running tap water. The sections were counterstained lightly in Gills haematoxylin between 10 and 60 s and then washed well in tap water. Finally, the sections were dehydrated through graded alcohols, cleared by xylene and sections mounted in DPX. For GLUT1 and CD31/34, microwave pretreatment was undertaken at the start of the procedure and GLUT1 was then visualised using the Envision HRP and DAB combination followed by CD31/34 using the Envision AP and Vector red combination as described above.

### Image capture

Images were captured using an Axioscope trans-illumination microscope (Zeiss) connected to a 3-CCD colour camera (JVC). Images were digitised with a Matrox Meteor frame grabber in a PCI bus 600 MHz Pentium desktop PC. Analysis was performed using routines developed in Visilog 5.02 software (Noesis Vision Inc, Lesvlis Cedex, France). Image-capture procedures were standardised for light intensity and background subtraction at different magnifications. There was no automated analysis of data, but simple tools (area measurement, line measurement, counting grids), developed in Visilog 5.02 software, were used to complement visual identification and scoring.

### Immunohistochemical analysis

As hypoxia-related markers tended to be expressed in contiguous cells, the area of pimonidazole, CAIX and GLUT1 staining was assessed at × 100 (× 10 eyepiece and objective) using a tool that enabled manual drawing of regions around the stained cells, the overall tumour area and necrosis; this made possible the exclusion of stroma. Two sections from each tumour were systematically assessed and between 3 and 45 fields were viewed for each specimen. The fraction of cells stained for hypoxia was expressed as a percentage of the total tumour area.

Geographic colocalisation was assessed semiquantitatively by visual inspection of stained areas in serial sections.

The proliferative index was assessed from Ki-67 staining in the retrospective series by computer-aided manual counting of multiple fields (× 400) captured from two sections of each tumour.

Vascularity was assessed by ‘hot spot’ counting and overall vascular density. The sections were systematically surveyed for a ‘hot spot’ of vessels using a × 5 objective. The ‘hot spot’ was then centralised in the microscope field, viewed under a final magnification of × 200 and individual vessels were counted. Between three and 10 hot spots were counted for each tumour, depending on the size of the section. The highest hot spot count was assigned to the tumour. Vascular density involved simply counting all vessels at × 100 magnification in multiple fields from two sections of each tumour. This represented a field size of 0.710 mm^2^. The vascularity was expressed as the number of vessels per mm^2^ of tissue section.

### Statistical analysis

The relationships between biological parameters (as continuous variables) and T stage and grade were tested using one-way analysis of variance and the *χ*^2^ test. The biological parameters were compared by either the Spearman rank correlation coefficient for continuous variables or by *χ*^2^ statistic. Survival intervals were calculated using the Kaplan–Meier product limit method. In the calculation of local recurrence-free survival and metastasis-free survival, nonfailures were censored at last follow-up or at death. For cause-specific survival, patients who died as a result of bladder cancer were classed as failures and nonfailures and were censored at death from other causes or at the last follow-up. For overall survival, patients who died of any cause were classed as failures and non-failures and were censored at the last follow-up. All time intervals were calculated from the date of first radiotherapy treatment. Individual factors were tested using the log rank test, where *P*<0.05 was considered significant. The Cox proportional hazard model was used for multiple regression analysis.

## RESULTS

### Staining patterns for pimonidazole, CAIX and GLUT1 in the prospective study

**Figure 1:**
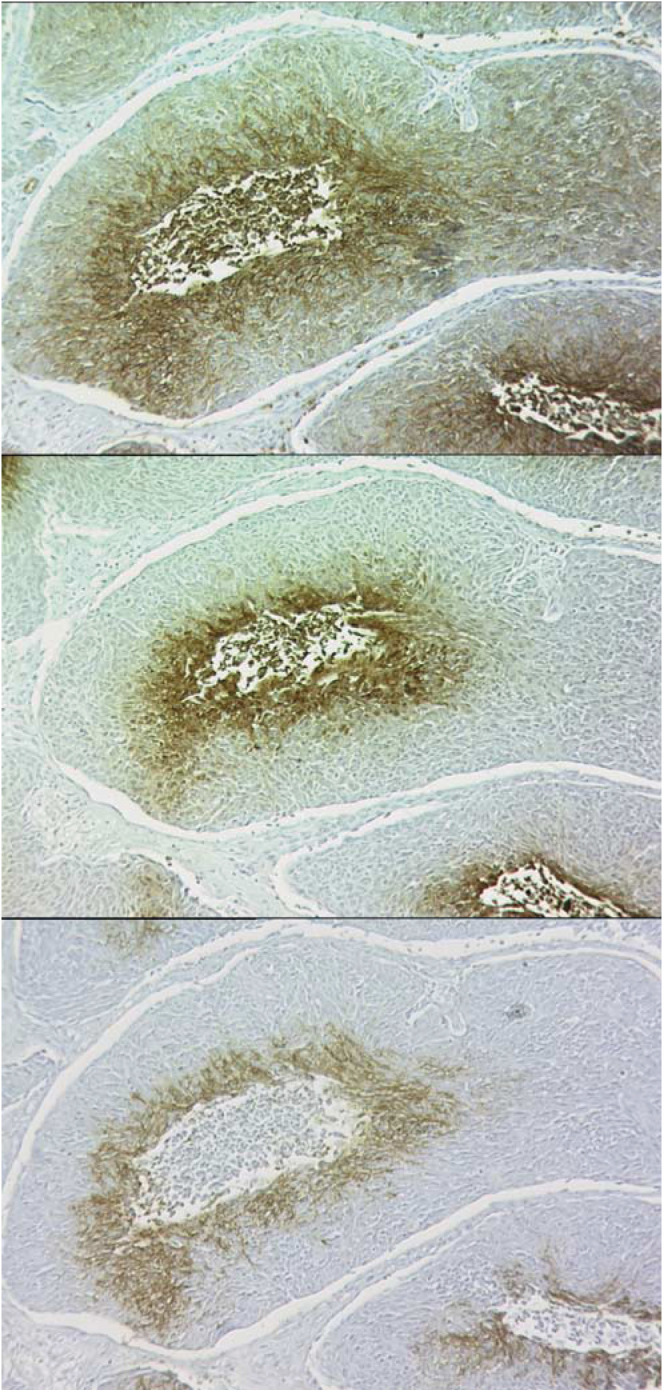
Serial sections stained for pimonidazole (top), GLUT1 (middle) and CAIX (bottom).

There was considerable intra- and intertumour variation in both the amount and intensity of staining for each of the three hypoxia-associated markers. Staining with pimonidazole was both cytoplasmic and nuclear and tended to be contiguous, but some focal hypoxia was noted, and even single-cell staining was seen. The darkest staining intensity was consistently seen adjacent to necrotic regions, but there were also other regions of dark staining unassociated with necrosis within tumour sections. CAIX and GLUT1 staining was predominantly membranous and was strongly associated with areas adjacent to necrosis and there was a paucity of staining near blood vessels. Geographical colocalisation of pimonidazole, CAIX and GLUT1 was assessed by visual inspection. The majority of tumours showed similar staining patterns for the three markers with the exception of one tumour that stained extensively for pimonidazole but minimally for both GLUT1 and CAIX, while another showed no pimonidazole binding but substantial GLUT1 and CAIX staining. Concordant localisation is exemplified in Figure 1

, which demonstrates closely matched areas where CAIX and GLUT1 showed identical expression within a region that bound pimonidazole. In this case, the binding of pimonidazole was more extensive than the intrinsic markers, but this was variable between tumours. Colocalisation was prevalent but there were also regions of mismatch, more often where pimonidazole and GLUT1 were both positive and CAIX was negative.

In the prospective series of patients, the median stained fraction and range of values for pimonidazole, GLUT1 and CAIX was 9% (0–38%), 15% (0–45%) and 12% (0–35%), respectively. Three tumours failed to demonstrate any pimonidazole binding, while only one was negative for GLUT1 and two did not express CAIX. The markers were all highly correlated with each other (Table 2

**Table 2:** Linear regression and correlation analysis of the intrinsic and extrinsic hypoxia markers in the prospective and retrospective studies

**Parameters**	**Slope**	**Intercept**	***r*^2^**	***P*-value**
Pimo *vs* CAIX	0.69	+4.8	0.74	<0.0001
Pimo *vs* GLUT1	0.76	+3.7	0.82	<0.0001
CAIX *vs* GLUT1 prospective	0.91	+1.2	0.91	<0.0001
CAIX *vs* GLUT1 retrospective	0.90	−0.8	0.96	<0.0001

Pimo=pimonidazole; CAIX=carbonic anhydrase IX; GLUT1=glucose transporter-1 protein.

, Figure 2

**Figure 2:**
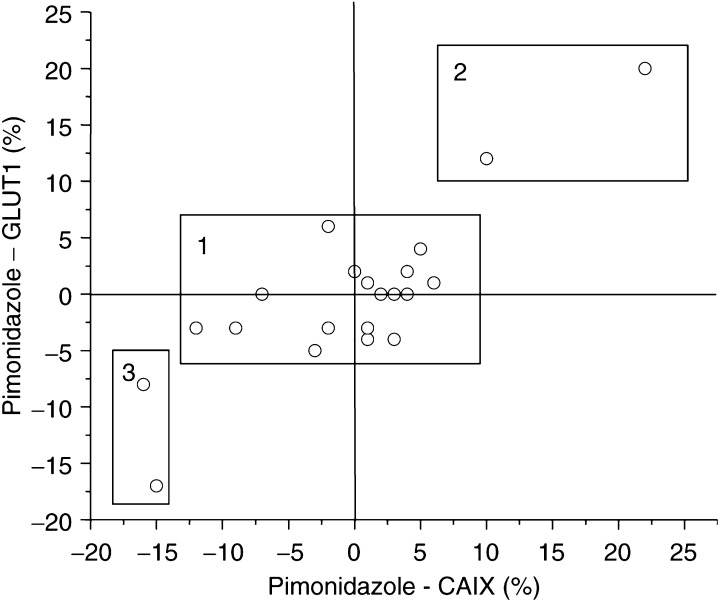
Comparison between intrinsic and extrinsic markers of hypoxia. The data are presented as the intrinsic markers subtracted from the pimonidazole score for each tumour in the prospective study. Box 1 indicates good absolute agreement, while boxes 2 and 3 represent disparity between the markers.

), but Figure 2 highlights that in box 2 there were tumours that showed expression of the extrinsic marker in the absence of the intrinsic markers and vice versa in box 3.

### Biological parameters in the retrospective study

In the retrospective group of patients the median hypoxic fraction as defined by GLUT1 was 6.5% (range 0–62%) compared to 3.5% (range 0–67%) with CAIX (Table 3

**Table 3:** Distribution of biological parameters in the retrospective study

**Parameter**	**Median (mean)**	**Range**	**CV**
GLUT1	3.5 (11.3)	0–67	129.4
CAIX	6.5 (9.4)	0–62	145.3
Ki-67	35.7 (34.9)	2.6–61.7	35.6
Hot spot	30 (31.5)	12–55	34.3
Vascular density	55.8 (66.4)	21–180	54.4

**Figure 3:**
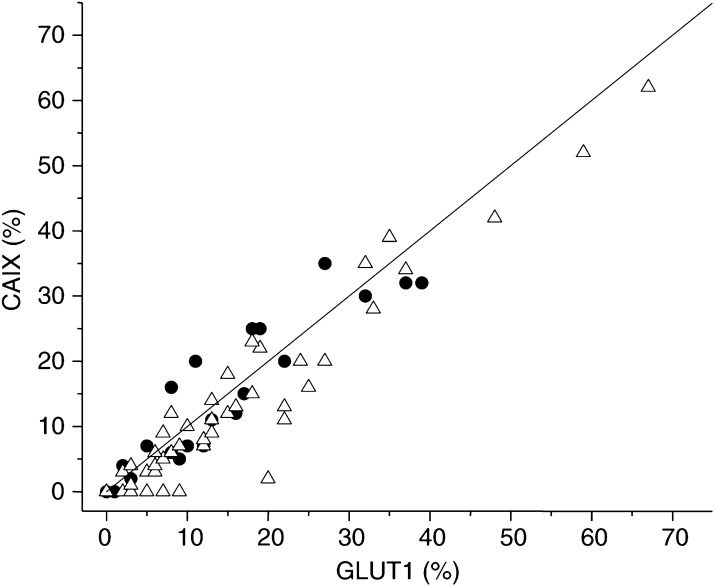
Correlation between GLUT1 and CAIX in the prospective (•) and retrospective (▵) studies.

GLUT1=glucose transporter-1 protein; CAIX=carbonic anhydrase IX; CV=coefficient of variation.

). Again, there was a strong correlation between CAIX and GLUT1 (Table 2, Figure 3

). However, there were differences between this series and the prospective patients notably in the proportion of tumours that showed no evidence of expression of the intrinsic markers. In this series, there were 22 (34%) tumours that were negative for both CAIX and GLUT1 and a further five were without CAIX staining, but had low levels of GLUT1 expression. Interestingly, the absolute levels of both CAIX and GLUT1 positivity was similar in both the prospective and retrospective series in those tumours which expressed the proteins (Figure 3).

Figure 4

**Figure 4:**
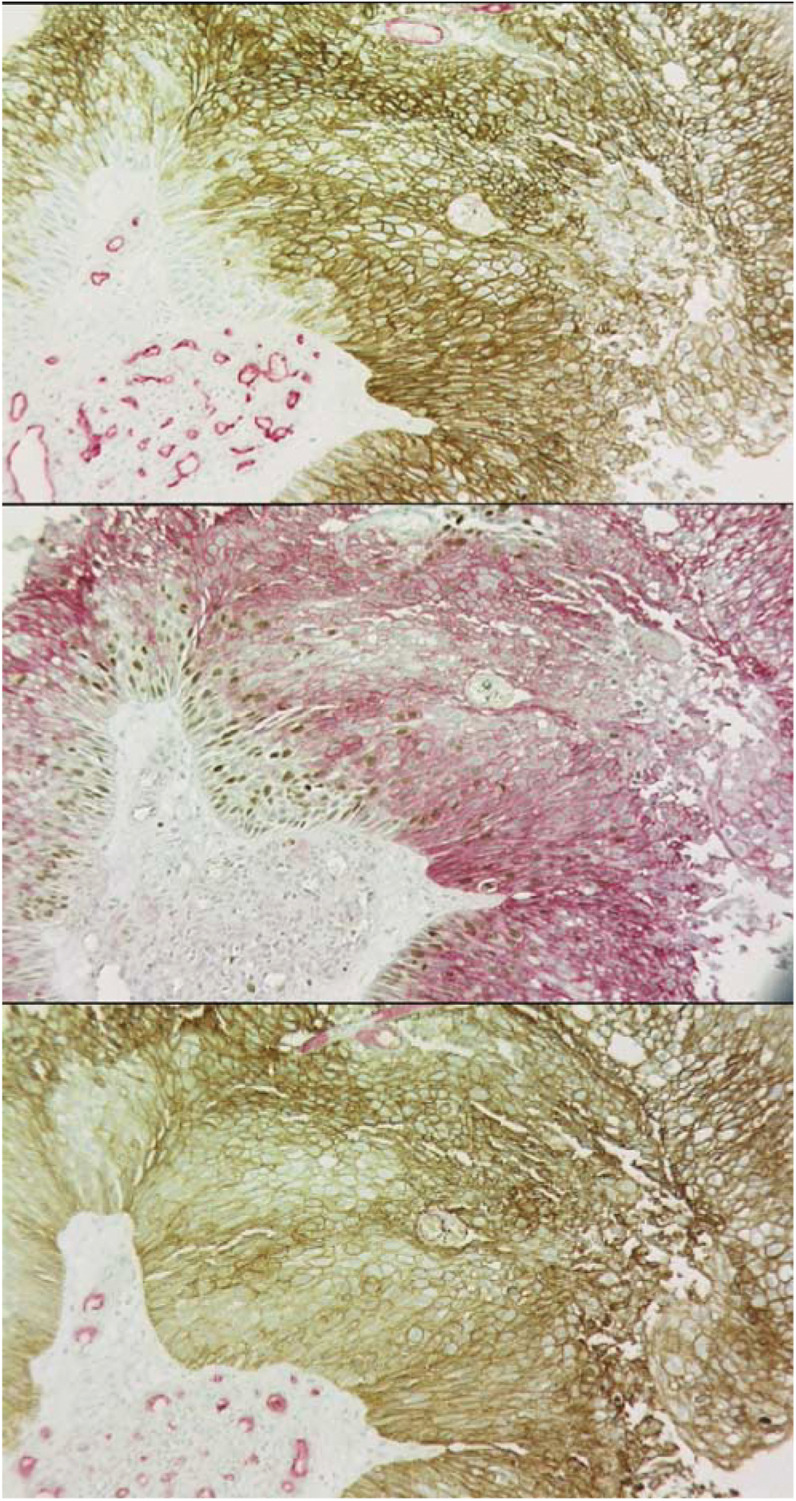
Examples of double staining. Serial sections showing similar areas stained for CAIX (brown) *vs* CD31/34 (red) (upper), CAIX (red) *vs* Ki-67 (brown) (middle), and GLUT1 (brown) *vs* CD31/34 (red) (lower).

shows examples of dual staining from which the data in Table 3 were obtained. In general, there was close geographical concordance of GLUT1 and CAIX as was noted in the prospective study. Although not a formal part of this study, it was noted that both CAIX and GLUT1 tended to stain closer to blood vessels than pimonidazole and that there was an overlap between these markers and Ki-67. The median positivity of 35.7% for Ki-67 represents a value that is indicative of rapid proliferation characteristics. Interestingly, although there was considerable variation in GLUT1 and CAIX both within and between individual tumours, proliferation and vascular parameters showed less variation. Correlation analysis had already revealed a highly significant association between CAIX and GLUT1 (Table 2), but there was no correlation between these markers and vascularity assessed by either vascular density or ‘hot spot’ analysis. However, there was a positive correlation between GLUT1 and Ki-67 (Spearman's correlation=0.294, *P*=0.018) and a near-significant correlation between CAIX and Ki-67 (Spearman's correlation=0.24, *P*=0.056). Figure 5

**Figure 5:**
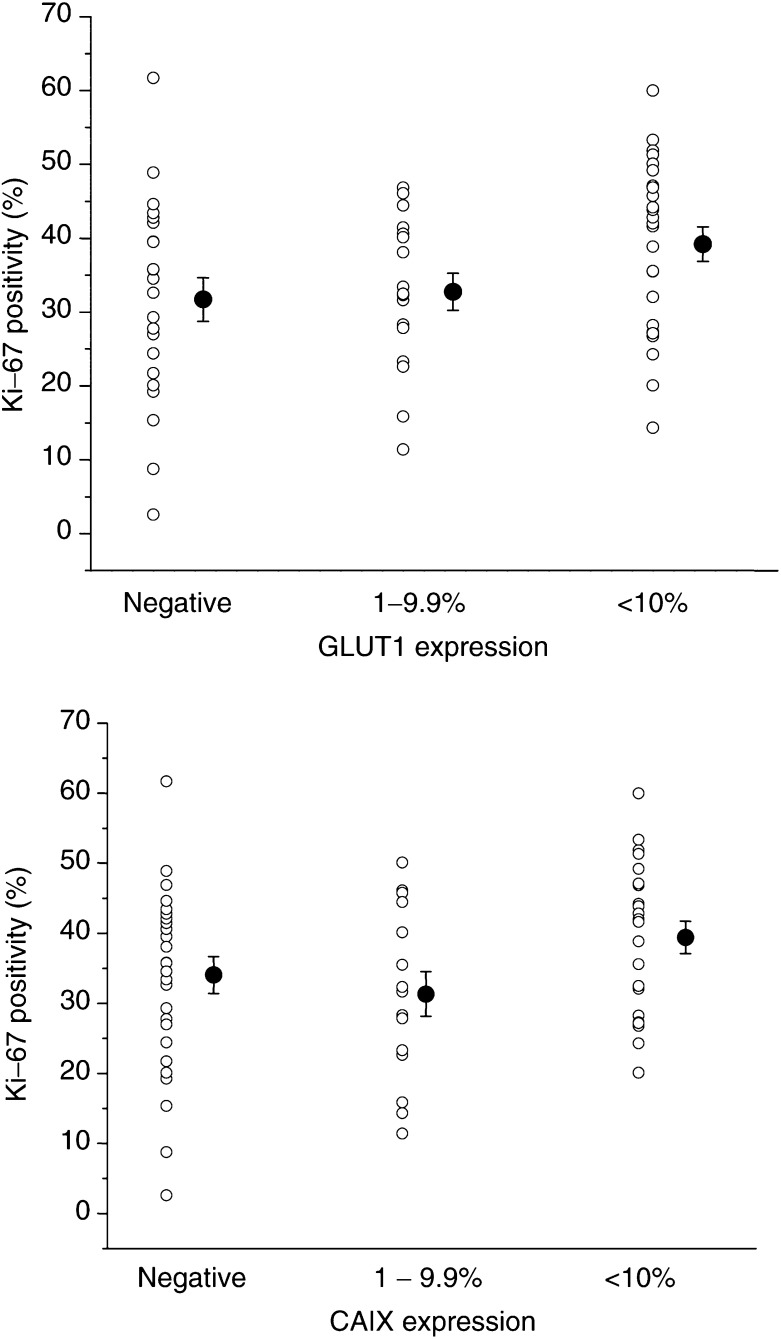
Relationship between the proliferation and expression of CAIX and GLUT1. The data show the individual values for Ki-67 positivity and the mean and s.e.m. for the three categories of intrinsic marker expression.

shows that this was not a strong relationship, but proliferation was significantly higher in those tumours with the highest levels (>10%) of both GLUT1 (*P*=0.025) and CAIX (*P*=0.029) compared to those tumours that were either negative or had low levels of expression. Vascularity showed no association with proliferation, but ‘hot spot’ count did correlate with overall vascular density (Spearman's correlation=0.65, *P*=0.0001).

### Correlation with clinicopathological features and outcome

None of the biological parameters, in either study, were related to disease stage, grade or patient age.

Table 4

**Table 4:** Univariate analysis of biological parameters in relation to various clinical endpoints

	**End point**							
	**Local control**		**Time to distant metastastis**		**Cause-specific survival**		**Overall survival**	
**Parameter**	***χ*^2^**	***P*-value**	***χ*^2^**	***P*-value**	***χ*^2^**	***P*-value**	***χ*^2^**	***P*-value**
GLUT1	1.56	0.21	0.22	0.64	5.84	0.016	8.06	0.0045
CAIX	0.82	0.36	0.29	0.59	4.16	0.041	8.57	0.0034
Ki-67	2.23	0.14	1.39	0.24	0.63	0.43	0.006	0.94
MVD	0.068	0.79	0.011	0.92	0.002	0.96	0.07	0.79
Hot spot	1.40	0.23	0.013	0.91	0.57	0.45	0.001	0.98

**Figure 6:**
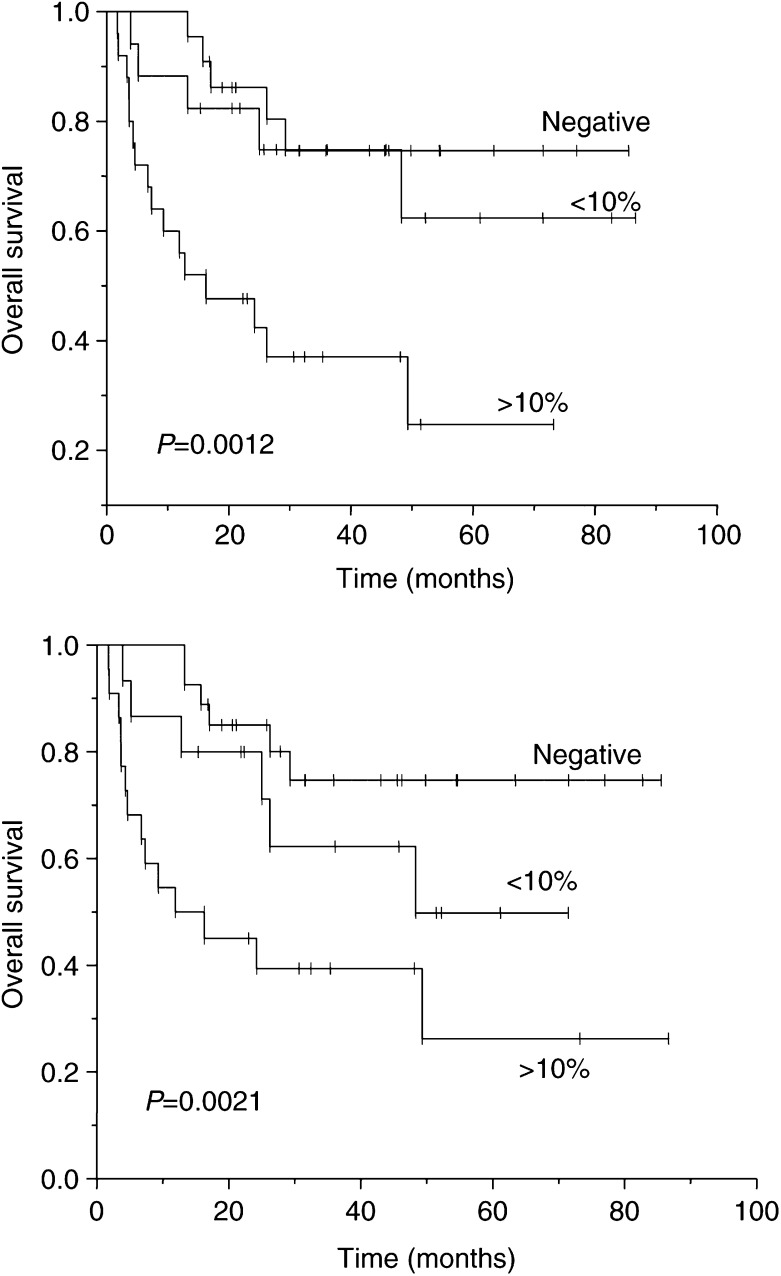
Influence of GLUT1 (upper panel) and CAIX (lower panel) on the overall survival of patients treated with radical radiotherapy, carbogen and nicotinamide. The data were classified into tumours with no expression of the proteins and using a cutoff of 10% for those that were positive.

GLUT1=glucose transporter-1 protein; CAIX=carbonic anhydrase IX. Each parameter was categorised according to the median value; MVD=mean vascular density.

shows the data obtained using the median value of each parameter as a cutoff for univariate analysis for a series of clinical outcome end points. None of the parameters had a significant influence on local control or time to metastasis; the only trend was that rapid proliferation was associated with worse local control. In contrast, there was a highly significant difference in the overall and cause-specific survival when patients were stratified according to the intrinsic markers of hypoxia. The 5-year overall survival rate in tumours expressing higher than median values of GLUT1 was 32% compared to 72% with low levels; the figures were 35% and 71% for CAIX. This analysis was taken a step further and the patients were delineated into three groups, those that were negative for either GLUT1 or CAIX and the positive tumours were stratified above or below a value of 10%. The data are presented in Figure 6

. These curves show that tumours without evidence of hypoxia-associated marker expression have the best survival prospects, suggesting that these are genuinely negative tumours and not attributable to sampling error. Low GLUT1 or CAIX is associated with intermediate survival, while 5-year survival rates of 25% were estimated for those patients in which either hypoxia surrogate marker exceeded 10%; this represented 29 (39%) and 22 (34%) patients for GLUT1 and CAIX, respectively.

Multivariate analysis was undertaken using the biological parameters as continuous variables in combination with age, T stage and grade; CAIX and GLUT1 were entered both individually and together. None of the parameters showed independent significance for local control. For both cause-specific and overall survivals, CAIX and GLUT1 showed high independent significance when entered into the analysis individually. The risk ratios (RR) for the overall survival were 3.21 (95% CI 1.16–10.22; *P*=0.02) for CAIX and 3.14 (95% CI 1.23–10.09; *P*=0.03) for GLUT1. Similar results were obtained for cause-specific survival. When both variables were entered into the analysis the significance was lost, CAIX 2.12 (95% CI 0.39–10.94); GLUT1 2.12 (95% CI 0.36–10.33), suggesting that they are interchangeable as prognostic markers and that either CAIX or GLUT1 is predictive, but this is not refined by the addition of the second marker.

## DISCUSSION

Hypoxia in tumours is primarily a pathophysiological consequence of a structurally and functionally compromised microcirculation and the deterioration of diffusion conditions. Hypoxia appears to be strongly associated with tumour propagation, malignant progression and resistance to therapy, and it has thus become a central issue in tumour physiology and cancer treatment (Brown, 2000; Hockel and Vaupel, 2001; Harris, 2002). An overview analysis showed that modification of tumour hypoxia significantly improved the locoregional tumor control after radiotherapy in head and neck (H&N) cancer and to a lesser extent in bladder tumours (Overgaard and Horsman, 1996). However, unlike H&N cancer (Nordsmark *et al*, 1996; Brizel *et al*, 1997), it was not possible to confirm the presence and clinical significance of hypoxia in bladder cancer due to the inaccessibility of the tumour site for oxygen electrode-based measurements. The availability of histological markers of hypoxia has widened the opportunity to study tumours in more remote sites. The binding of pimonidazole adducts is perhaps the most robust and widely accepted marker of cellular hypoxia (Varia *et al*, 1998; Nordsmark *et al*, 2001; Raleigh *et al*, 2001; Kaanders *et al*, 2002). However, the technique is invasive as it requires an injection of pimonidazole to be scheduled prior to surgical procedures. To circumvent this, there has been a growing interest in studying hypoxia-regulated proteins as potential surrogates for the more invasive techniques. These have included HIF-1*α* and HIF-2*α* (Beasley *et al*, 2002), VEGF (Salven *et al*, 1997), GLUT1 and GLUT3 (Younes *et al*, 1997; Airley *et al*, 2001; Baer *et al*, 2002) and CAIX (Loncaster *et al*, 2001; Swinson *et al*, 2003). In the prospective arm of this study, we have demonstrated that pimonidazole, GLUT1 and CAIX show a high degree of overlap in their tumour localisation pattern and a significant correlation in their interpatient comparison. In addition, in the retrospective study of patients treated by ARCO(N), both GLUT1 and CAIX overexpression were significantly associated with worse cause-specific and overall survivals but not local control.

Validation of proteins such as CAIX or GLUT1 as intrinsic markers of cellular hypoxia is clearly important to facilitate the routine study of tumour hypoxia in clinical trials and ultimately to use this to direct clinical practice outside trials. Several studies have attempted to achieve this goal. Airley *et al* (2003) undertook a direct comparison of all three markers in cervix cancer specimens. The study concluded that there were similar staining patterns for all three markers and that they were significantly correlated with each other, although this comparison was made on a semiquantitative scoring system. Other studies have shown a weak but significant correlation between GLUT1 and *p*O_2_ values in cervix cancer (Airley *et al*, 2001), a strong correlation between CAIX and *p*O_2_ values in cervix cancer (Loncaster *et al*, 2001) and a weak correlation between CAIX and pimonidazole in H&N cancer (Kaanders *et al*, 2002). The general consensus from these studies is that expression of both CAIX and GLUT1 might be potential surrogates for hypoxia. Interestingly, CAIX was predictive of both disease-free and metastasis-free control but not local control, while GLUT1 only had a significant association with metastasis-free survival in similar cervix cancer patients (Airley *et al*, 2001; Loncaster *et al*, 2001). CAIX was without clinical significance in H&N cancer in contrast to pimonidazole binding that predicted for locoregional control and disease-free survival (Kaanders *et al*, 2002).

In bladder cancer, we have previously shown concordance between CAIX and pimonidazole staining patterns (Wykoff *et al*, 2000). Other studies have demonstrated that GLUT1 expression is not seen in normal bladder mucosa, but is present in malignant bladder mucosa with greater expression seen in muscle-invasive tumours compared to superficial bladder cancer (Chang *et al*, 2000). In this study, there was no correlation between recurrence and GLUT1 expression. CAIX expression was noted to be greater in superficial disease compared to invasive disease (Turner *et al*, 2002) and to show siginificant overlap with VEGF mRNA expression; CAIX did not predict outcome in superficial disease. In contrast, another study of patients treated by cystectomy for muscle-invasive bladder cancer found a significantly worse overall survival if they had >10% GLUT1-stained fraction compared to those with less staining (Younes *et al*, 2001).

This study demonstrates that bladder cancer is a tumour with an average hypoxic fraction of around 10% and we have now shown that intrinsic markers of hypoxia, both GLUT1 and CAIX, can be used to predict survival. Accordingly, bladder cancer can be seen alongside cervical cancer, H&N cancer and soft-tissue sarcomas as a tumour in which hypoxia is a predictive factor for survival. No correlation was seen, however, between hypoxia and local control or metastases-free survival. This finding may be explained by the fact that all tumours in this group were treated using a hypoxia-modifying regimen that included the administration of carbogen and nicotinamide (Hoskin *et al*, 1997; Hoskin *et al*, 1999). As a consequence, any negative influence of hypoxia on the outcome from radiotherapy may have been overcome.

In this series, there was a weak correlation between the Ki-67 index and local control. Other studies that have used Ki-67 as a marker of proliferation in transitional cell bladder cancer have reported a significantly lower progression-free and disease-specific survival rates in patients with higher Ki-67 indices (Popov *et al*, 1997; Lara *et al*, 1998; Moonen *et al*, 1998a; Rodel *et al*, 2000). Clinical data relating the effect of treatment duration on bladder outcome are equivocal (Maciejewski and Majewski, 1991; De Neve *et al*, 1995; Moonen *et al*, 1998b).

The data from this study establish that muscle-invasive bladder cancer is a relatively rapidly proliferating, hypoxic tumour where a rationale for alternative treatments such as ARCON is justified. To maximise the benefit of ARCON, it may be possible to consider individualisation of treatment on the basis of intrinsic immunohistochemical markers of hypoxia and proliferation, which can be performed on routine paraffin-embedded biopsy material. Validation of such an approach requires evaluation in a randomised cohort of patients that is currently being undertaken in the multicentre BCON trial of carbogen and nicotinamide in bladder cancer.
